# N‐Acetylcysteine Reduces Alveolar Bone Loss and Mitigates Systemic Oxidative Damage in Rats With Apical Periodontitis

**DOI:** 10.1111/iej.70108

**Published:** 2026-02-05

**Authors:** Ian Wesley Rocha dos Santos, Deiweson Souza‐Monteiro, Deborah Ribeiro Frazão, Zuleni Alexandre Lisboa da Silva, João Daniel Mendonça de Moura, Jorddy Neves Cruz, Fabrício Mezzomo Collares, Renata Duarte de Souza‐Rodrigues, Luciano Tavares Ângelo Cintra, Rafael Rodrigues Lima

**Affiliations:** ^1^ Laboratory of Functional and Structural Biology, Institute of Biological Sciences Federal University of Pará Belém Brazil; ^2^ Dental Materials Laboratory, Department of Conservative Dentistry, School of Dentistry Federal University of Rio Grande do Sul Porto Alegre Brazil; ^3^ Department of Preventive and Restorative Dentistry School of Dentistry of Araçatuba–UNESP–Universidade Estadual Paulista São Paulo Brazil

**Keywords:** alveolar bone loss, animal model, antioxidant, oxidative stress, periapical periodontitis

## Abstract

**Aim:**

This study aimed to evaluate the effects of N‐acetylcysteine (NAC) supplementation in apical periodontitis (AP) induced in rats.

**Methodology:**

Eighteen male Wistar rats were randomly assigned to three groups: control, AP, and AP plus NAC. NAC was administered by oral gavage (100 mg/kg/day), beginning 1 day after lesion induction and continued daily until the day preceding euthanasia. AP induction was performed by exposing the dental pulp of the lower first molars bilaterally, maintaining this condition for 28 days. After this period, the animals were euthanized, and the following biological materials were collected: blood (for systemic oxidative stress analysis) and hemimandibles for histopathological and histochemical, and micro‐computed tomography analyses, aiming to measure bone quality parameters and periapical volume. Statistical analyses were performed using one‐way ANOVA and Tukey's post hoc test. In addition, correlation analyses and multivariate analyses of variance (MANOVA) were performed on the biochemical parameters.

**Results:**

The study results showed that animals supplemented with NAC had greater preservation of bone quality parameters and a reduction in periapical volume progression when compared to the only apical periodontitis group. Additionally, in the analysis of systemic oxidative stress, supplemented animals showed higher antioxidant parameter levels and lower oxidant levels compared to non‐supplemented animals, which also showed reduced preservation of bone collagen content.

**Conclusions:**

The study findings suggest that NAC supplementation promoted greater preservation of bone quality, reduced periapical volume development, and modulation of endogenous antioxidant and oxidant aspects. This indicates that NAC can decrease local and systemic damage caused by AP, highlighting its potential as an adjunctive agent in processes involving systemic oxidative stress and the preservation of biological structures.

## 1 Introduction

Apical periodontitis (AP) is characterised as a chronic inflammatory disease often associated with the infectious process of the root canals, leading to the loss of tissues associated with these structures, including the periodontium and periapex (Tibúrcio‐Machado et al. 2021; Domenico Ricucci and Siqueira 2010). Approximately 50% of the global adult population has at least one tooth affected by AP (Tibúrcio‐Machado et al. 2021). Due to this factor, understanding the perpetuating conditions of this disease and identifying effective treatments are of utmost importance, especially treatments or supplements that may assist in achieving a more favourable and essential prognosis.

Endodontic treatment remains the gold standard for managing apical periodontitis, offering predictable outcomes for disease eradication. Although treatment is expected to lead to a cure, persistent risks of ongoing damage persist due to the oxidative and inflammatory processes associated with the disease, such as bone resorption and loss of periodontal ligaments (Siqueira et al. 2014; Wen et al. 2024). Importantly, contemporary endodontics must consider the systemic implications of apical periodontitis (Frazão et al. 2023). Clinical evidence suggests that apical periodontitis triggers substantial systemic oxidative stress in humans (Matos‐Sousa, Chemelo, et al. 2024; Matos‐Sousa, Souza‐Monteiro, et al. 2024), thereby establishing this phenomenon as a well‐established clinical concern. These findings underscore the need for adjunctive therapeutic strategies targeting both local and systemic manifestations of the disease.

Antioxidants are utilised as complementary treatments for various conditions, including apical periodontitis. Consequently, antioxidants such as N‐acetylcysteine (NAC) have garnered growing research interest to assess their effects on oxidative processes. These effects include boosting endogenous antioxidant levels, stabilising reactive oxygen species (ROS), and improving cellular defences (Şehirli et al. 2023; Wen et al. 2024).

Accordingly, NAC acts as a precursor for glutathione synthesis, one of the main endogenous antioxidants responsible for neutralising ROS and maintaining cellular redox homeostasis. Upon conversion to cysteine, NAC provides the essential substrate for glutathione biosynthesis, which also involves glutamate and glycine in ATP‐dependent enzymatic reactions. Supplemental administration of NAC increases plasma cysteine levels, thereby enhancing intracellular glutathione production, particularly in erythrocytes. This process plays a crucial role in protecting cells against oxidative stress and modulating inflammatory responses (Zhang et al. 2022; Du et al. 2022; Yan et al. 2020). Moreover, studies have demonstrated that NAC exerts beneficial effects on bone remodelling and osteogenic differentiation by reducing lipopolysaccharide‐induced osteoclastogenesis and restoring the balance between bone resorption and formation (Xi et al. 2022; Yan et al. 2020). In the endodontic context, evidence indicates that NAC possesses significant antibacterial and anti‐inflammatory activity, being capable of enhancing conventional therapies and promoting tissue regeneration (Abdulrab et al. 2022; Şehirli et al. 2023).

The objective of this study was to evaluate the impact of NAC administration on AP, with a focus on its antioxidant activity within the inflammatory process and its systemic biochemical implications. Through tissue and biochemical analyses, we aimed to determine whether NAC can mitigate damage such as bone resorption and destruction of periodontal ligament structures, as well as the systemic effects induced by apical periodontitis.

## 2 Methods

**FIGURE 1:**
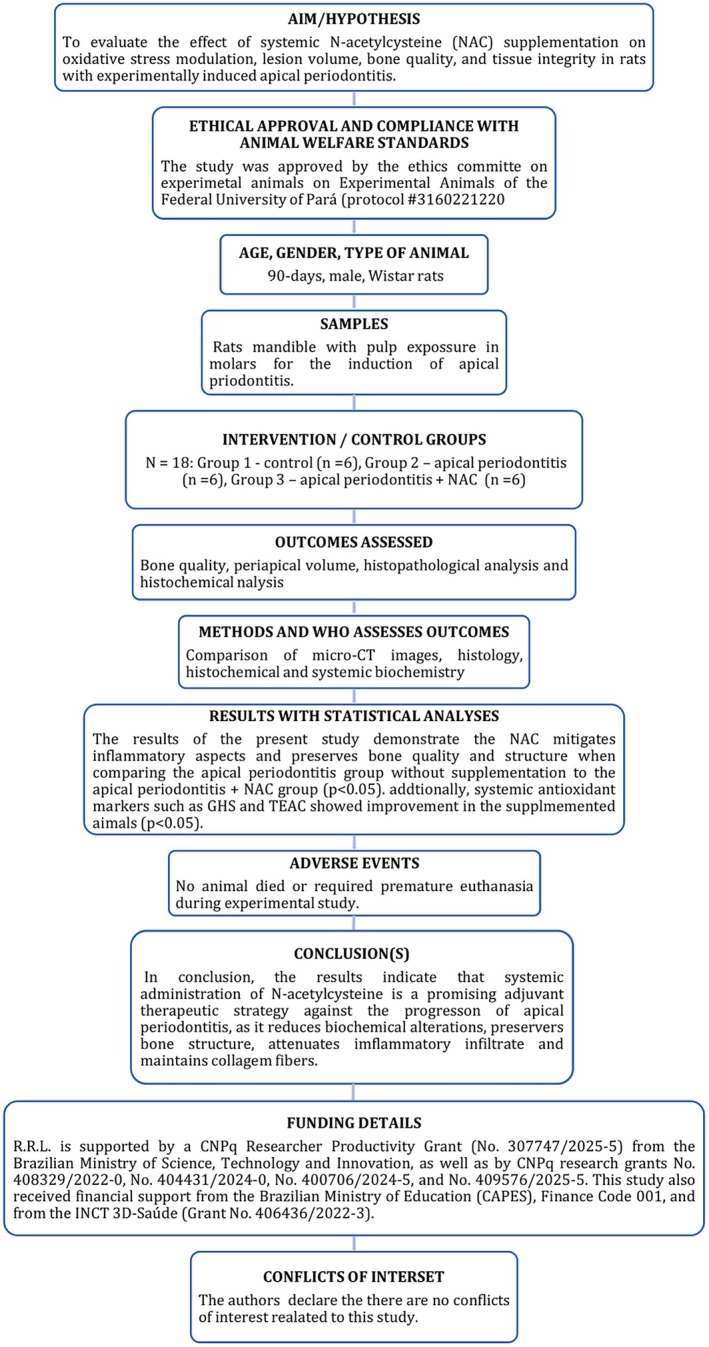
Flowchart outlining the steps of the study following PRIASE guidelines.

The study followed the guidelines established by the Preferred Reporting Tems for Animal Studies in Endodontology (PRIASE) (Nagendrababu et al. 2021). The study flowchart is presented in Figure 1.

### 2.1 PRIASE Flowchart for Essential Steps for Reporting Animal Studies

The PRIASE 2021 flowchart, depicted in Figure 1, includes 12 domains outlining the essential steps for reporting animal studies.

All procedures were approved by the Ethics Committee on Animal Experimentation (CEUA: No. 3160221220). Additionally, the study was conducted following the recommendations of the Guide for the Care and Use of Laboratory Animals (2011), the ARRIVE guidelines (Percie du Sert et al. 2020).

As inclusion criteria, we considered the absence of visible pathologies during veterinary examination, normal weight gain during acclimatisation, and successful induction of AP (confirmed by standardised pulp exposure protocol in the AP groups and morphological findings). Animals in the control group maintained a healthy periodontal status throughout the study. No exclusions were necessary as all subjects met health criteria, and all surgical procedures were completed without complications.

### 2.2 Animal Experimentation and Experimental Groups

Eighteen male Wistar rats ( *Rattus norvegicus* ), aged 90 days, were housed in polypropylene cages (30 cm × 20 cm × 12 cm) with a maximum of four animals per cage to minimise overcrowding, with *ad libitum* access to food and water. They were housed under a 12‐h light/dark cycle and maintained at a controlled temperature of 25°C ± 1°C. Environmental conditions were rigorously controlled, with a 12‐h light/dark cycle (lights activated at 7:00 AM), a stable room temperature of 25°C ± 1°C, and relative humidity maintained at 50% ± 10%. Bedding material, consisting of wood shavings, was replaced once a week to ensure optimal hygiene standards.

The animals were randomly assigned into three groups (*n* = 6/group): control group (CTL), comprising animals that did not undergo the lesion induction procedure; apical periodontitis (AP) group, consisting of animals that underwent access surgery without subsequent NAC supplementation and apical periodontitis + NAC (AP + NAC) group, which included animals subjected to access surgery for lesion induction and subsequently supplemented with NAC. A random number generator (https://www.random.org/) was used to ensure unbiased group assignment. This procedure was conducted by two authors, who were aware of group allocations due to their involvement in the experimental setup. The sample size was determined using G*Power software (Statistical Power Analysis 3.1.9.4) based on the effect size reported by Matos‐Sousa, Chemelo, et al. (2024) and Matos‐Sousa, Souza‐Monteiro, et al. (2024). Parameters were set at an effect size of 𝑓 = 1.107, a Type I error rate (α) of 0.05, and a power of 0.95, which indicated a sample size of 5 animals per group, yielding a power of 0.968. To account for potential sample loss during the experiment while maintaining statistical power, the sample size was increased to 6 animals per group.

### 2.3 Apical Periodontitis Induction

Following established protocols (Frazão et al. 2023; Matos‐Sousa, Chemelo, et al. 2024; Matos‐Sousa, Souza‐Monteiro, et al. 2024; de Moura et al. 2025), the animals were subjected to intraperitoneal anaesthesia (2% xylazine hydrochloride, 8 mg/kg + 10% ketamine hydrochloride, 90 mg/kg; confirmed by loss of paw withdrawal and corneal reflexes). After this, the pulp of the left and right first molars was exposed using a #1/4 carbide drill for low rotation (KaVo Dental, Biberach an der Riß, Germany) coupled to the X‐Smart Plus motor (Dentsply Maillefer, Ballaigues, Switzerland) at a speed of 1200 rpm and a torque of 4.0 Ncm. Access was made in the distal fossette of the first molars to a depth equivalent to the active tip of the drill.

Post‐surgical analgesia was administered via subcutaneous dipyrone (100 mg/kg/day for 3 days), a regimen selected for its demonstrated efficacy in rodent models and compliance with established veterinary pain management protocols (Lupu et al. 2022). Comprehensive monitoring included twice‐daily assessments for pain indicators (reduced mobility, altered grooming patterns) and surgical site infections. Weekly body weight measurements were systematically recorded to track health status throughout the study.

All animals remained free from adverse events, with none meeting predefined humane endpoint criteria (> 20% weight loss, persistent anorexia, or severe lethargy). Animal care was supervised by qualified personnel holding advanced degrees (MSc/PhD) in biomedical sciences and certified training in laboratory animal welfare, ensuring adherence to ethical standards during all procedures.

### 2.4 N‐Acetylcysteine Antioxidant Activity

#### 2.4.1 Determination of the Trolox Equivalent Antioxidant Capacity (TEAC)

Prior to initiating the experiment, we evaluated the antioxidant capacity of NAC. This was achieved using the ABTS^•+^ [2,2′‐Azino‐bis (3‐ethylbenzothiazoline‐6‐sulfonic acid)] and DPPH (2,2‐diphenyl‐1‐picrylhydrazyl) assays. These assays measure antioxidant potential in terms of their equivalence to Trolox (6‐hydroxy‐2,5,7,8‐tetramethylchromono‐2‐carboxylic acid), a potent antioxidant and a water‐soluble synthetic vitamin E analog.

#### 2.4.2 ABTS

The ABTS^•+^ radical scavenging assay was determined according to the methodology adapted from (Miller et al. 1993) and modified by (Re et al. 1999). ABTS^•+^ was prepared using 7 mM ABTS^•+^, and 140 mM of potassium persulfate (K_2_O_8_S_2_) incubated at room temperature without light for 16 h. Then, the solution was diluted with phosphate‐buffered saline until it reached an absorbance of 0.700 (± 0.02) at 734 nm.

The synthetic antioxidant Trolox was used as a standard solution for the calibration curve. To measure the antioxidant capacity, 2.97 mL of the ABTS^•+^ solution was transferred to the cuvette, and the absorbance at 734 nm was determined using an Nm Kasvi spectrophotometer. Then, 0.03 mL of the sample was added to the cuvette containing the ABTS^•+^ radical and after 5 min, the second reading was performed. The results were expressed as mM. The values found for the samples were compared to the Trolox standard (1 mM).

#### 2.4.3 DPPH

This method was performed to analyse the potential of NAC to inhibit the 1,1‐diphenyl‐2‐picrylhydrazyl (DPPH•) radical, a violet chromophore, resulting in the formation of the hydrogenated DPPH product, which is yellow or colourless. The test was conducted according to the method proposed by Blois (1958). To measure the antioxidant capacity, the initial absorbance of a 0.1 mM DPPH• solution diluted in ethanol was determined. Subsequently, 0.6 mL of DPPH• solution, 0.35 mL of distilled water, and 0.05 mL of the sample were mixed and placed in a water bath at 37°C for 30 min. After that, the absorbances were determined using a spectrophotometer (Nm Kasvi) at 517 nm. Trolox served as the standard for the calibration curve. Results were expressed in mM and compared to the 1 mM Trolox standard.

### 2.5 N‐Acetylcysteine Supplementation

After solubilising NAC in distilled water, the animals underwent orogastric gavage (100 mg/kg/day) (Abdel‐Wahab and Moussa 2019; Akgun et al. 2005), starting on the first day after lesion induction and continuing daily until the day before euthanasia, for a total of 28 days. This procedure was carried out daily at the same time throughout the experiment, maintaining this protocol until the euthanasia of the animals. The animals were weighed weekly to adjust the dose.

### 2.6 Biological Samples Collection

Following 28 days of supplementation, animals were humanely euthanized under deep anaesthesia (ketamine/xylazine 90/9 mg/kg i.p.) to ensure complete pain elimination prior to procedures, confirmed by loss of paws withdrawal and corneal reflexes. For blood collection, cardiac puncture was performed using heparinized syringes after confirming the absence of pedal reflexes, with 5 mL samples centrifuged at 3000 *g* for 10 min at 4°C. The plasma was then aliquoted into pre‐chilled polypropylene tubes and flash‐frozen at −80°C. To minimise tissue degradation artefacts, immediate transcardial perfusion was conducted using heparinized saline (0.9% NaCl with 10 IU/mL heparin) followed by 4% formaldehyde. Mandibular dissection employed sterile surgical instruments (15 scalpel and curved Mayo scissors) by trained personnel to prevent tissue trauma, with specimens divided for micro‐CT and histopathology analyses.

### 2.7 Biochemical Analysis

Systemic oxidative stress biomarkers were quantified to evaluate the peripheral repercussions of apical periodontitis and the potential antioxidant effects of NAC, as local inflammatory processes can induce measurable redox imbalances in circulation (Matos‐Sousa, Chemelo, et al. 2024; Matos‐Sousa, Souza‐Monteiro, et al. 2024). The collected blood underwent centrifugation to isolate plasma, which was subsequently utilised for biochemical analyses to assess the antioxidant activity of N‐acetylcysteine in plasma. To evaluate antioxidant activity, TEAC (using the same method previously described in Section 2.4.1) and reduced glutathione (GSH) levels were measured, both of which are classified as antioxidant parameters. As a pro‐oxidant marker indicative of oxidative stress, the concentration of TBARS was determined.

#### 2.7.1 Reduced Glutathione

The concentrations of GSH were determined using the Ellman method (Ellman 1959). This technique is based on the ability of GSH to reduce 5,5′‐dithiobis (2‐nitrobenzoic acid) (DTNB, Sigma‐Aldrich D8130) to 5‐thio‐2‐nitrobenzoic acid (TNB). Quantification was performed by spectrophotometry at a wavelength of 412 nm. GSH concentrations were expressed as μmol/mL.

#### 2.7.2 Thiobarbituric Acid Reactive Substances

The determination of TBARS, a method for evaluating lipid peroxidation, was performed using the procedure described by Kohn and Liversedge (1944). This technique is based on the reaction of malondialdehyde (MDA) and other substances with thiobarbituric acid (TBA; Sigma‐Aldrich T5500) at pH 2.5 and 94°C, forming the MDA‐TBA complex, which exhibits a pink colour with absorbance at 535 nm. Since the reaction is not specific to MDA, it is understood that TBA can also react with sugars, amino acids, proteins, and bilirubin (Heliana Gomes et al. 2003). Elevated TBARS concentrations are used as indicators of oxidative stress.

The technical procedure for the method involved the initial preparation of potassium monobasic phosphate (KH_2_PO_4_, 75 mM, Synth, 35210) in distilled water (pH 2.5). This solution was used to prepare the TBA solution (10 mM). A 100 μL sample aliquot was added to 500 μL of the 10 mM TBA solution. The mixture was then incubated in a water bath at 94°C for 60 min. After incubation, the sample was cooled for 10 min, followed by the addition of 4.0 mL of 1‐butanol. The mixture was vigorously vortexed and centrifuged at 25 000 rpm for 10 min. Finally, 1.0 mL of the supernatant was collected for spectrophotometric reading at 535 nm. MDA standard (1,1,3,3‐tetrahydroxypropane, Sigma‐Aldrich T9889) was used, and results were expressed in nmol/mL.

### 2.8 Micro‐Computed Tomography

For microtomographic evaluation, the hemimandible was fixed in 10% formaldehyde. The hemimandibles were then subjected to micro‐computed tomography (MicroCT.SMX‐90 CT; Shimadzu Corp., Kyoto, Japan). Images were captured with a 360° rotation using an intensity of 70 kV and 100 mA. The images were subsequently reconstructed using the inspeXio SMX‐90CT software (Shimadzu Corp., Kyoto, Japan), with a voxel size of 10 μm and a resolution of 1024 × 1024, resulting in 541 images per sample. All datasets were exported in Digital Imaging and Communications in Medicine (DICOM) format.

The alveolar bone region was reconstructed and analysed using CTAn software (version 1.15.4.0; Bruker, Kontich, Belgium). To ensure consistency, the hemimandibles were standardised during positioning, with an emphasis on the periodontal ligament space in the selected sections. Following established methodologies in the literature (Frazão et al. 2023; Matos‐Sousa, Chemelo, et al. 2024; Matos‐Sousa, Souza‐Monteiro, et al. 2024; Chen, Chen, et al. 2019), the volume of interest (VOI) was defined to include the ligament space and/or the region of bone destruction around the tooth roots. For each sample, the examiner, who was calibrated and blinded to the groups, manually outlined the area of destruction surrounding the first mandibular molar, starting at the mesial root and extending to the distal root, and concluding when the second molar became visible.

In addition to quantifying periapical volume using the CTAn software, further assessments were conducted to evaluate the quality of the alveolar bone. This analysis utilised a set of 50 images of the bone surrounding the first mandibular molar. The region of interest (ROI) was identified as the alveolar bone encompassing the roots of the molar. The examiner manually delineated the bone in each coronal section, starting from the proximal side of the mesial root and extending to the distal side of the distal root. To differentiate between cortical bone, trabecular bone, and bone marrow, a standardised threshold range (32–82) was applied to the images. Bone quality was assessed based on the following parameters: trabecular thickness (Tb.Th), trabecular number per millimetre (Tb.N), and trabecular spacing (Tb.Sp). These parameters were measured within the unaffected bone regions, excluding areas impacted by the lesion.

### 2.9 Histological Analysis

The other hemimandibles were decalcified in EDTA, then dehydrated in ethanol at sequential concentrations (70%, 80%, 90%, Absolute I, and Absolute II), followed by treatment with xylene and embedding in paraffin. After embedding, the samples were sectioned using a microtome (Leica Microsystems, Nussloch, Germany), resulting in 6 sections of 5 μm thickness per animal, which were mounted on individual slides. Three of these sections were stained with haematoxylin and eosin (H&E), while the remaining three were stained with PicroSirius Red.

Images from H&E slides were captured using a colour digital camera (DS‐Fi3, Nikon, Tokyo, Japan) coupled to a microscope (Nikon Eclipse Ci‐S, Tokyo, Japan) to characterise the bone preservation and inflammation in the groups. PicroSirius Red‐stained images were obtained using the same microscope system, equipped with a polarising filter module, at 40× magnification, which enabled the evaluation of the collagen content in the remaining alveolar bone tissue (Chemelo et al. 2022). The area of collagen fibres was measured using ImageJ software, with the arithmetic mean of the area calculated from three fields/sections per sample. The results were expressed in μm^2^.

### 2.10 Statistical Analysis

Statistical analysis was performed using GraphPad Prism 9.0 software (GraphPad Software Inc., La Jolla, CA, USA). Data normality was assessed using the Shapiro–Wilk test, considering *p* > 0.05. For group comparisons, one‐way ANOVA followed by Tukey's post hoc test was applied. Moreover, correlation analysis and multivariate analyses of variance (MANOVA) were performed on the biochemical data using Jamovi version 2.3 (www.jamovi.org ‐ The jamovi project, 2025) . The significance level of *p* < 0.05 was used to determine statistically significant differences. Results are presented as mean ± standard error of the mean (SEM).

## 3 Results

### 3.1 N‐Acetylcysteine Showed Antioxidant Effects on Biochemical Assays

The results of the antioxidant analyses demonstrated the potential of N‐acetylcysteine. In the ABTS assay, NAC exhibited consistent antioxidant activity. Complementarily, the DPPH assay, known for its higher sensitivity, indicated that NAC's antioxidant capacity was comparable to that of the TROLOX standard (Table 1). These findings confirm the functionality of the drug used in this study.

**TABLE 1:** Values of mean and standard deviation (SD) obtained from the analyses performed on the substance (N‐acetylcysteine) using the 2,2′‐azino‐bis‐3‐ethylbenzothiazoline‐6‐sulfonic acid (ABTS) and 1,1‐diphenyl‐2‐picrylhydrazyl (DPPH) methods.

	N‐acetylcysteine	
	Mean	SD
DPPH (mM)	0.910	0.027
ABTS (mM)	0.320	0.004

### 3.2 N‐Acetylcysteine Promoted Antioxidant Effects on the Systemic Oxidative Stress Triggered by Apical Periodontitis

**FIGURE 2:**
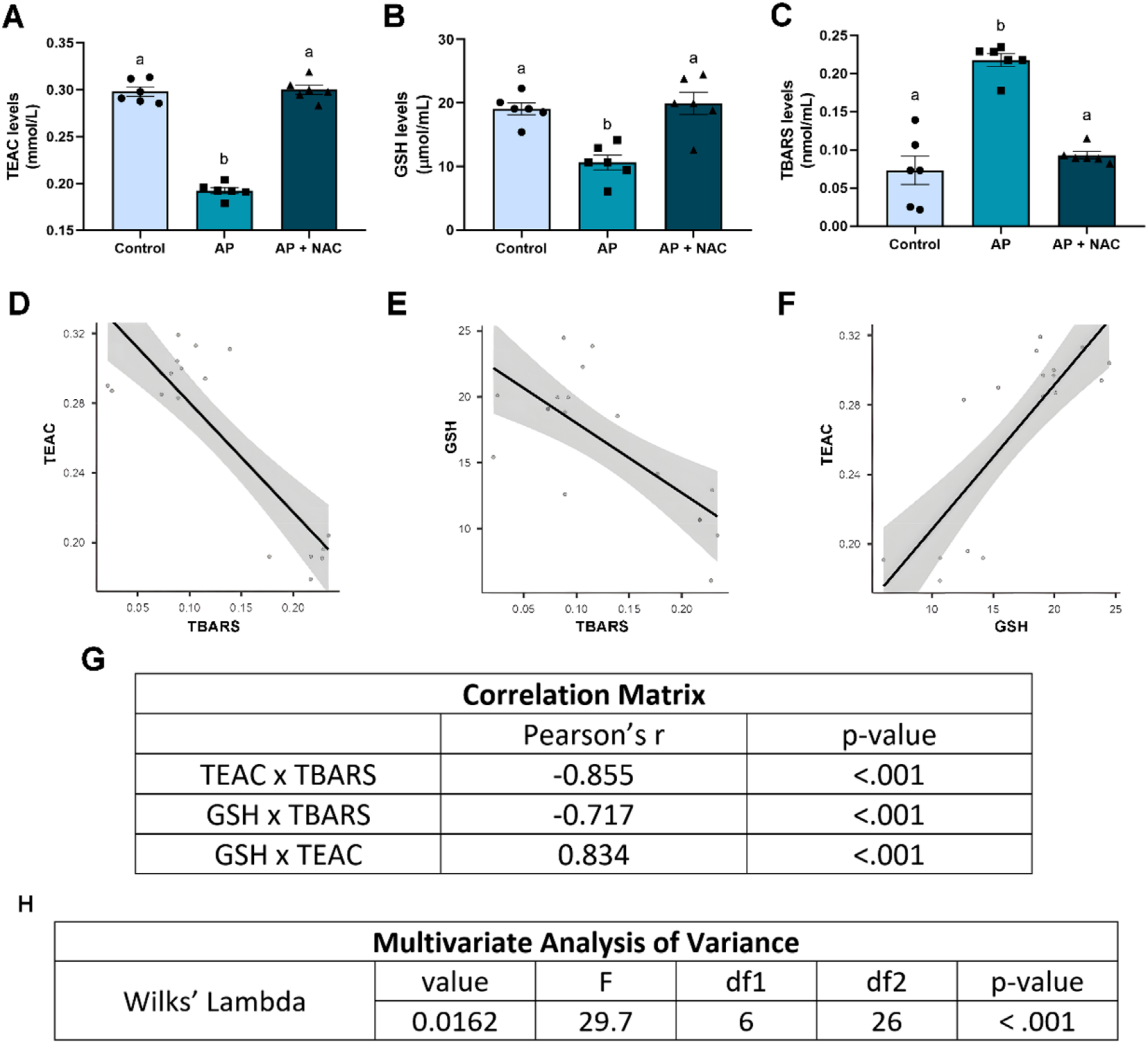
Systemic biochemical analysis of rats with apical periodontitis supplemented with NAC (100 mg/kg/day) during 28 days. Trolox Equivalent Antioxidant Capacity levels (A, TEAC), thiobarbituric acid reactive substances levels (B, TBARS), reduced glutathione levels (C, GSH). Results are expressed as mean ± standard error of the mean. One‐way ANOVA with Hockey's post hoc test considering *p* < 0.05 was adopted, and similar overlined letters indicate the absence of statistical difference (*n* = 6/group). Correlation analysis between TEAC versus TBARS results (D), GSH versus TBARS results (E), GSH versus TEAC results (F), and Correlation Matrix with Pearson's *r* and *p* values of all tests (G). Multivariate Analysis of Variance using Wilks' Lambda with value, *F* (test statistic), df1 (degree of freedom 1), df2 (degree of freedom 2), and *p* values (H).

The assessment of oxidative biochemistry showed that the AP + NAC group exhibited higher levels of total antioxidant capacity when compared to the AP group (AP + NAC: 0.30 ± 0.004 mmol/mL, AP: 0.192 ± 0.003 mmol/mL, adjusted *p*‐value < 0.0001), with similar levels to the CTL group (CTL: 0.29 ± 0.004 mmol/mL, adjusted *p*‐value = 0.94) (Figure 2A).

In addition, the evaluation of GSH also showed improvement caused by NAC in this antioxidant parameter when compared to non‐supplemented animals (AP + NAC: 19.94 ± 1.74 μmol/mL, AP: 10.64 ± 1.15 μmol/mL; adjusted *p*‐value = 0.0004), with similar levels to the CTL group again (CTL: 19.08 ± 0.91 μmol/mL, adjusted *p*‐value = 0.89) (Figure 2B).

Our lipid peroxidation analysis, measured by TBARS levels as a pro‐oxidant parameter, showed that the AP + NAC group exhibited lower levels when compared to the AP group (AP + NAC: 0.092 ± 0.004 nmol/mL, AP: 0.217 ± 0.008 nmol/mL, adjusted *p*‐value < 0.0001). Furthermore, when compared to the CTL group, the supplemented group did not show a statistically significant difference (CTL: 0.073 ± 0.018 nmol/mL, adjusted *p*‐value = 0.49) (Figure 2C).

The correlation analysis revealed a significant correlation among all three comparisons: TEAC versus TBARS, GSH versus TBARS, and TEAC versus GSH (*p* < 0.001, Figure 2D–G). The analysis revealed a strong inverse correlation for TEAC versus TBARS (Pearson's *r*: −0.855) and GSH versus TBARS (Pearson's *r*: −0.717), while a strong direct correlation was observed in TEAC versus GSH (Pearson's r: 0.834).

Finally, the MANOVA results are shown in Figure 2H, and the Wilks' Lambda test revealed a significant difference (*F*: 29.7, *p* < 0.001) between the compared groups and all biochemical variables.

### 3.3 N‐Acetylcysteine Modulated Alveolar Bone Loss in Micro‐CT Analysis

**FIGURE 3:**
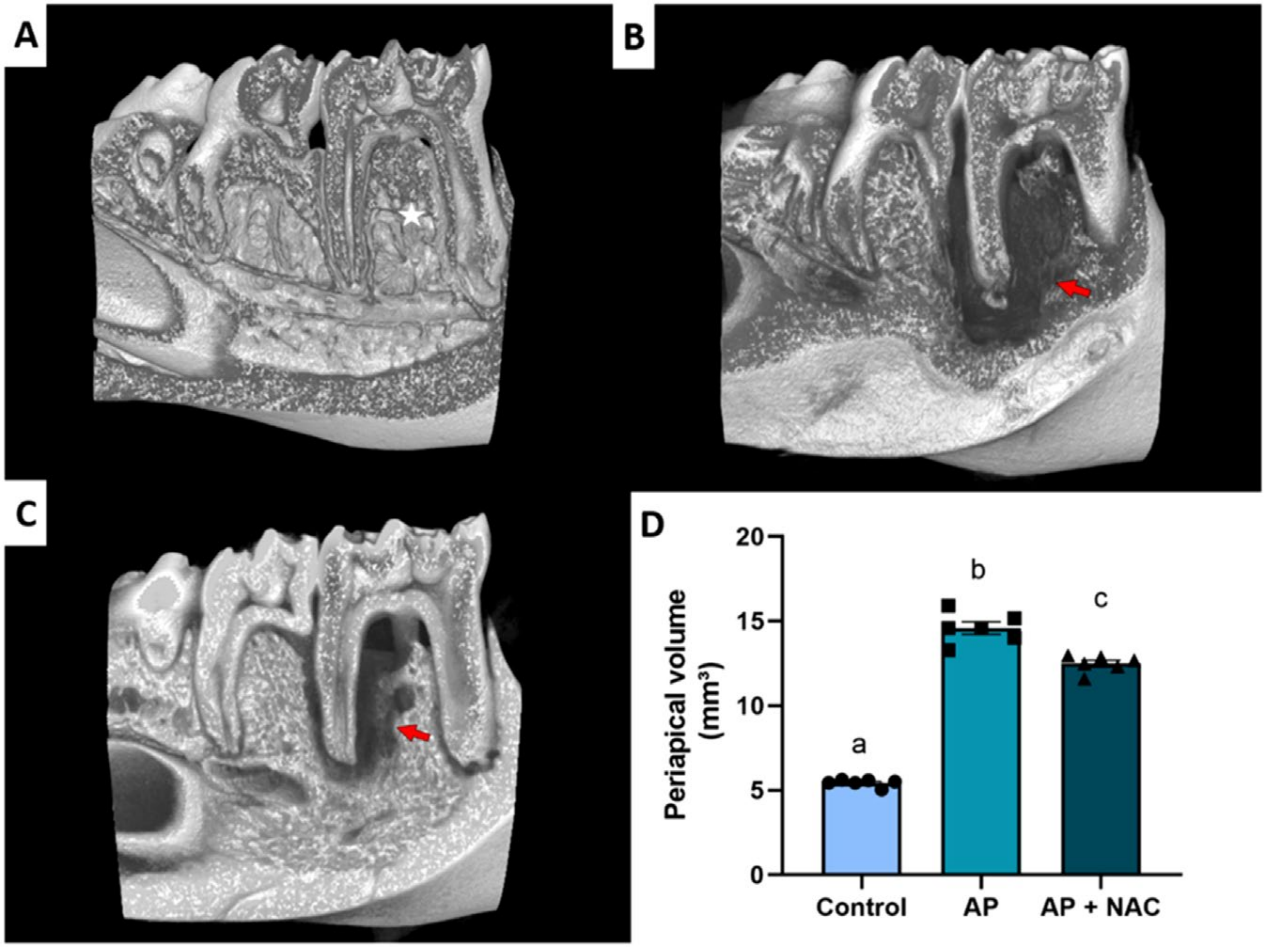
Periapical volume evaluation of rats supplemented with N‐acetylcysteine. CTL group (A), AP group (B), and AP + NAC group (C). Red arrows indicate periapical lesions; the interradicular bone exhibits a normal pattern, as indicated by the white star. The graph presents the periapical volume measurement. Data are presented as mean ± standard deviation, with statistical significance denoted by different letters above the bars (*p* < 0.05) (*n* = 6/group).

In the evaluation of periapical volume, it was confirmed that the AP group (AP: 14.59 ± 1.177 mm^3^) had a larger periapical volume compared to the CTL group (CTL: 5.443 ± 0.2729 mm^3^; *p* < 0.0001). However, the AP + NAC group (AP + NAC: 12.69 ± 1.006 mm^3^) attenuated the periapical volume compared to the AP group (*p* = 0.0387) (Figure 3).

**FIGURE 4:**
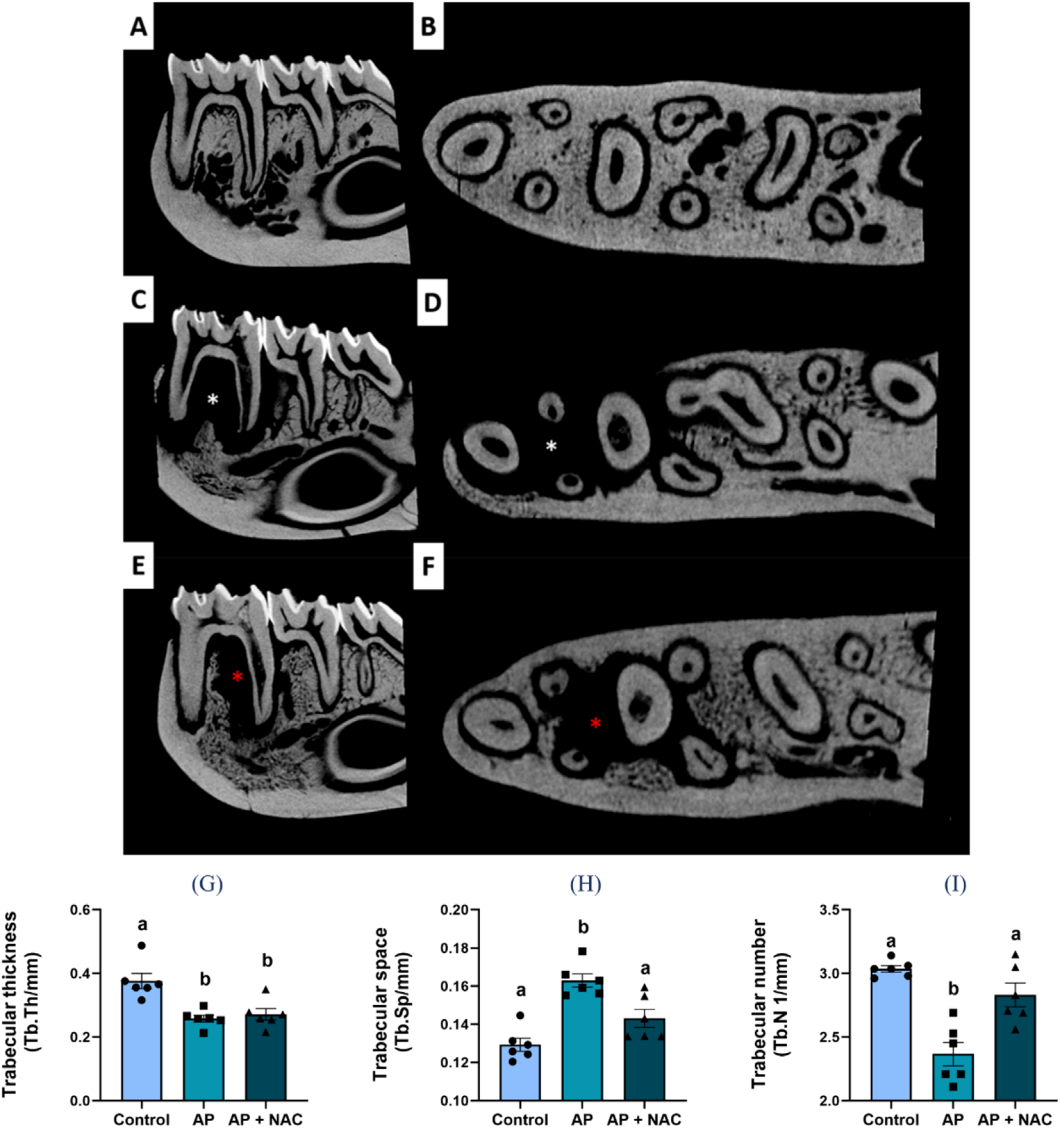
N‐acetylcysteine supplementation effects on bone quality of rats with apical periodontitis. The 2D reconstructed images correspond to the CTL group (A, B), the apical periodontitis group (B, C), and the AP + NAC group (E, F). The white asterisks indicate the lesion area in the AP group, while the red asterisks highlight the lesion area in the AP + NAC group. The graphs present the following parameters: Trabecular thickness (Tb.Th, mm) (G), trabecular space (Tb.Sp, mm) (H), and trabecular number (Tb.N, 1/mm) (I). Data are presented as mean ± standard deviation, with statistical significance denoted by different letters above the bars (*p* < 0.05) (*n* = 6/group).

Regarding the alveolar bone quality, the evaluation of trabecular thickness (Tb.Th) showed no statistical differences between the AP + NAC group (0.2714 ± 0.017 mm) and AP (0.2578 ± 0.011 mm, *p* = 0.8617). Both the AP + NAC group and the AP group (0.2578 ± 0.011 mm) exhibited lower Tb.Th levels compared to the CTL group (0.3760 ± 0.023 mm; *p* < 0.005) (Figure 4).

The analysis of trabecular spacing (Tb.Sp) revealed that the AP group exhibited a significant increase in Tb.Sp (0.1629 ± 0.003 mm) compared to the CTL group (0.1293 ± 0.003; *p* < 0.0001). Conversely, the AP + NAC group (0.1431 ± 0.004 mm) demonstrated a reduction in Tb.Sp relative to the AP group (0.1629 ± 0.003 mm; *p* = 0.007) (Figure 4).

The analysis of trabecular number (Tb.N) showed that the AP group had a significantly lower Tb.N (2.366 ± 0.091 per mm) compared to the CTL group (3.035 ± 0.026 per mm; *p* < 0.0001). In contrast, the AP + NAC group exhibited an increase in Tb.Sp (2.831 ± 0.092 per mm) compared to the AP group (*p* = 0.0018) (Figure 4).

### 3.4 Animals Non‐Supplemented With N‐Acetylcysteine Showed More Periodontal Damage

**FIGURE 5:**
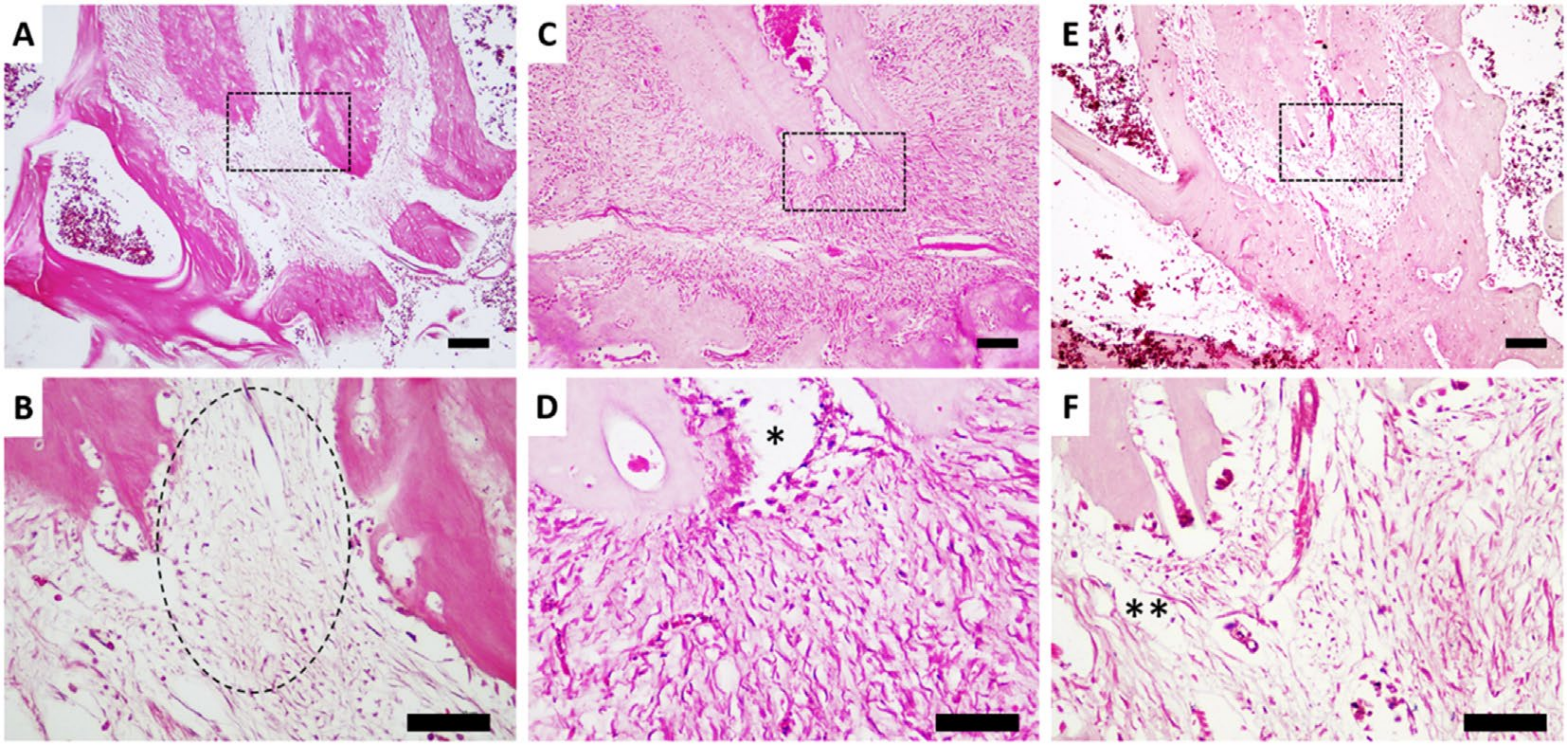
Histopathology of the periodontium of rats with apical periodontitis supplemented with N‐acetylcysteine (100 mg/kg). The groups are organised as follows: (A, B) CTL group, (C, D) AP group, and (E, F) AP + NAC group. Images (A, C, E) are shown with a 100 μm scale bar, whereas images (B, D, F) are shown with a 40 μm scale bar. The areas outlined by dotted squares highlight the root apex for better visualisation of histological changes. The dotted circle in image (B) indicates a healthy region at the pulp–periodontal ligament interface; in image (D), the asterisk marks an area of greater tissue loss associated with the periapical lesion; and in image (F), where two asterisks are shown, a smaller area of tissue discontinuity can be observed, indicating better preservation of the periodontal structures.

Histopathological analysis revealed that the AP + NAC group exhibited greater bone preservation, more organised connective tissue, and a reduced inflammatory infiltrate compared to the AP group. The latter showed marked destruction of the alveolar bone, loose and disorganised connective tissue, and significant inflammatory infiltrate. Additionally, the CTL group, representing healthy interradicular and periapical tissue conditions, displayed intact bone structures, dense and organised connective tissue, and the absence of inflammatory infiltrate (Figure 5).

### 3.5 N‐Acetylcysteine Supplementation Was Able to Mitigate the Apical Periodontitis Damage on Collagen Fibres of the Alveolar Bone

**FIGURE 6:**
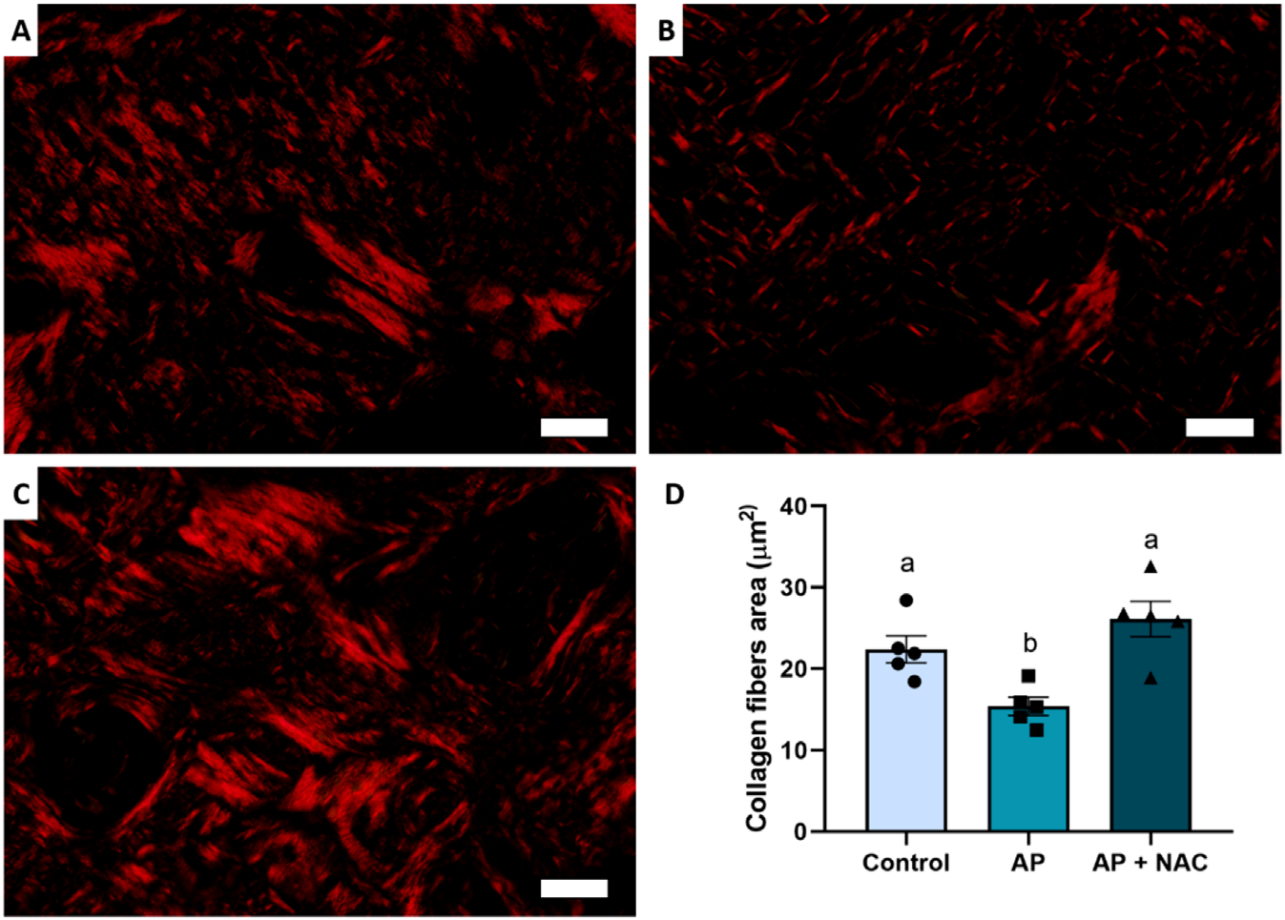
N‐acetylcysteine effects on collagen fibres of alveolar bone of rats with apical periodontitis. CTL group (A), AP group (B), and AP + NAC group (C). The graph illustrates the area of collagen fibres. Data are presented as mean ± standard deviation, with statistical significance indicated by different letters above the bars (*p* < 0.05) (*n* = 5/group). Scale bar = 20 μm.

Analysis of collagen fibres revealed that N‐acetylcysteine attenuated the damage caused by apical periodontitis. The AP + NAC group (24.36 ± 6.15 μm^2^) exhibited greater preservation of collagen fibres compared to the untreated AP group (14.03 ± 3.98 μm^2^), with levels very close to those of the CTL group (27.10 ± 8.68 μm^2^) (Figure 6).

## 4 Discussion

Our results demonstrated that NAC administration could attenuate systemic oxidative stress triggered by apical periodontitis through the modulation of blood antioxidant and pro‐oxidant parameters. It was found that NAC supplementation significantly increased the total antioxidant capacity and the levels of GSH while reducing the levels of TBARS, indicating a mitigation of oxidative stress. Moreover, NAC was also able to modulate alveolar bone loss, showcasing its potential to preserve bone structure and reduce the inflammatory damage caused by apical periodontitis. Histological analyses revealed that NAC supplementation resulted in greater organisation of connective tissue and a substantial reduction in inflammatory infiltrate compared to the AP group. Additionally, NAC effectively mitigated damage to collagen fibres, exhibiting collagen preservation levels close to those of the control group. Overall, these findings suggest that systemic administration of NAC may offer a promising therapeutic strategy in mitigating the pathological mechanisms of apical periodontitis progression.

Animal models have been used to mimic pathologies and their effects that commonly affect humans, such as apical periodontitis. Rats were selected as the experimental model due to their well‐documented anatomical and microbiological similarities to humans in apical periodontitis studies, alongside practical advantages such as standardised housing and cost‐effectiveness (Oz and Puleo 2011). Although no animal model fully replicates human disease pathogenesis, the 28‐day induction period was chosen based on prior validation by Frazão et al. (2023), which demonstrated consistent periapical lesion development and significant oxidative imbalance at 28 days. Systemic oxidative stress was quantified in blood to align with clinical evidence that associates apical periodontitis with elevated circulating oxidative biomarkers in humans (Matos‐Sousa, Chemelo, et al. 2024; Matos‐Sousa, Souza‐Monteiro, et al. 2024). This approach ensures translational relevance, as systemic oxidative imbalance reflects both local inflammatory progression and potential systemic sequelae of the disease.

In general, the use of antioxidants in the adjunctive treatment of periodontitis is based on evidence that supplementation with these components can help reduce periodontal damage and its systemic effects (Castro et al. 2019). A recent study examined the effects of various nutritional supplements, including probiotics, propolis, vitamin E, and vegetable oils, on the host response to inflammation and bone loss associated with experimental apical periodontitis. The findings concluded that these systemic nutritional supplements can effectively modulate host responses by reducing inflammation and bone resorption through biochemical, histological, and immunohistochemical pathways (Çankaya et al. 2025).

Our biochemical analyses showed that NAC supplementation significantly enhanced the antioxidant defence system, evidenced by higher TAC and GSH levels, and reduced lipid peroxidation, indicated by lower TBARS levels compared to the non‐supplemented group. High levels of GSH are beneficial because they play a fundamental role in various chemical and enzymatic reactions. Since NAC is a precursor to glutathione, it can be incorporated into cells, where it helps neutralise reactive oxygen species. This process reduces the cumulative effects of oxidative stress (Ross 1988). This systemic biochemical modulation correlates with attenuated alveolar bone loss in micro‐CT analysis, suggesting that NAC's regulation of redox homeostasis may protect against oxidative stress‐mediated bone resorption.

Micro‐CT analysis showed that the AP group exhibited significantly larger periapical volumes compared to the control group, confirming the pathological effects of apical periodontitis on periodontal tissues. In contrast, the AP + NAC group exhibited reduced periapical volumes, suggesting NAC attenuated disease progression. These findings align with the established role of oxidative stress in apical periodontitis pathogenesis (Frazão et al. 2023), where systemic oxidative stress is proportional to bone damage severity. Critically, our study advances Frazão et al.'s (2023) findings by demonstrating that antioxidant administration, such as NAC, can simultaneously reduce systemic oxidative stress and minimise alveolar bone damage, offering a dual therapeutic approach to modulate disease progression. The observed bone preservation likely stems from NAC's cysteine‐derived thiol group, which directly scavenges reactive oxygen species (Samuni et al. 2013).

The selected dose of N‐acetylcysteine (100 mg/kg/day) was based on established findings from rat models of inflammation and oxidative stress. In acetic acid‐induced colitis, this dose demonstrated significant reductions in inflammatory markers such as myeloperoxidase and oxidative damage by restoring glutathione and NO levels (Akgun et al. 2005). Similarly, in cisplatin‐induced neurotoxicity, 100 mg/kg/day NAC mitigated cerebral oxidative stress and pro‐inflammatory cytokines, including TNF‐α, IL‐1β, and IL‐6, while enhancing antioxidant enzymes like SOD and catalase (Abdel‐Wahab and Moussa 2019). Considering the shared oxidative and inflammatory pathways in apical periodontitis, this dosage was considered appropriate for addressing both local bone loss and systemic redox imbalance.

In terms of bone quality, our micro‐CT evaluation of trabecular thickness (Tb.Th), spacing (Tb.Sp), and number (Tb.N) highlighted the effects of apical periodontitis and the potential benefits of NAC. The results for Tb.Th revealed no significant differences between the AP and AP + NAC groups, with both groups showing lower values compared to the CTL group. This finding is consistent with previous studies where trabecular thickness was less sensitive to changes in inflammatory bone diseases, as seen in chronic apical periodontitis models (Chen, Lei, et al. 2019). On the other hand, the analysis of Tb.Sp and Tb.N demonstrated significant alterations due to the disease. The AP group exhibited a marked increase in Tb.Sp and a decrease in Tb.N compared to the control group, indicating deterioration of the trabecular bone structure. These findings are consistent with the pathophysiological understanding of apical periodontitis, where inflammatory cytokines and matrix metalloproteinases promote bone resorption and disrupt the bone microarchitecture (Aksoy et al. 2019). Notably, the AP + NAC group exhibited significant improvements in both Tb.Sp and Tb.N when compared to the AP group, with a reduction in Tb.Sp and an increase in Tb.N, suggesting that NAC may effectively counteract the detrimental effects of apical periodontitis on bone microstructure. These results align with the hypothesis that NAC's antioxidative properties may support the preservation of bone integrity in the presence of chronic inflammation, as seen in other inflammatory bone diseases (Yamada et al. 2010).

Our histological analysis reinforces the inflammatory and resorptive effects of AP on alveolar bone, as previously described. The presence of inflammatory infiltrate, loose and disorganised connective tissue, and bone degradation observed in our study are hallmark features of AP‐induced damage (Hernández‐Ríos et al. 2017; Conti et al. 2020). However, the magnitude attenuation of these histopathological alterations in the NAC‐supplemented group, as depicted in Figure 6, suggests a protective role of this antioxidant in preserving alveolar bone integrity at the microscope level. This action is likely mediated by the modulation of inflammatory responses and oxidative stress pathways involved in AP pathogenesis.

Furthermore, a key finding of our histological analysis was the impact of NAC on collagen fibre preservation in the alveolar bone microenvironment. Collagen fibres are essential for maintaining the structural and mechanical properties of bone and connective tissues, and their degradation is a major consequence of AP‐induced inflammation (Viguet‐Carrin et al. 2006; Matos‐Sousa, Chemelo, et al. 2024; Matos‐Sousa, Souza‐Monteiro, et al. 2024). The significant reduction in collagen fibres area observed in the untreated AP group highlights the destructive nature of the disease. However, NAC supplementation led to a substantial improvement in collagen fibre preservation, with values approaching those of the control group. These findings indicate that NAC may mitigate AP‐associated extracellular matrix degradation, potentially through its antioxidant properties, which have been reported to counteract reactive oxygen species (ROS)‐mediated tissue damage (Sun et al. 2014).

Given this, the mechanism of action of NAC has been described in several ways. Therefore, certain factors must be taken into consideration, including the dosage, route of administration, and the etiopathogenesis of the disease under investigation. Its activity in oxidative stress control models occurs through the increased bioavailability of cysteine, an amino acid essential for the synthesis of glutathione (GSH), a major endogenous neutralizer of reactive oxygen species (ROS) (Sahasrabudhe et al. 2023). Cysteine combines with glutamate to form γ‐glutamylcysteine, which is subsequently converted into GSH through the addition of glycine. GSH plays a crucial role in maintaining the cellular redox balance by neutralising ROS through electron donation, thereby stabilising these reactive molecules and restoring redox equilibrium. This antioxidant action mitigates the tissue damage induced by ROS during various inflammatory processes, including the chronic progression of diseases such as apical periodontitis. Moreover, NAC is thought to modulate inflammatory signalling pathways by inhibiting and reducing the recruitment of inflammatory cells, regulating protein kinases, and controlling the activity of nuclear factor kappa B (NF‐κB), a key regulator of innate and adaptive immune responses (Sahasrabudhe et al. 2023; Paschalis et al. 2018). When bone tissue damage is investigated, both local and systemic biochemical imbalances can be observed, generating factors capable of compromising the inflammatory response (Frazão et al. 2023). In this context, our results demonstrate that NAC administration effectively attenuated systemic biochemical imbalances, which was directly associated with reduced local damage, as evidenced by the micro‐CT data (Figure 4) showing greater preservation of bone architecture (Figure 5). This suggests an attempt to reestablish redox homeostasis through the suppression of the inflammatory response, further supported by the histological findings (Figure 6), which revealed a milder inflammatory infiltrate and improved tissue preservation.

While this study provides novel perspectives into NAC systemic and osteoprotective effects in apical periodontitis, some limitations should be considered. First, the NAC supplementation was conducted without endodontic therapy, which precludes direct translation to clinical scenarios where mechanical debridement is standard. While NAC dosage was selected based on prior rodent studies, pharmacokinetic profiling (e.g., bioavailability, tissue‐specific accumulation) was not performed, complicating human dose extrapolation. Besides, although micro‐CT and histopathology revealed reduced periapical volumes, molecular mechanisms (e.g., RANKL/OPG ratio, NF‐κB signalling, TRAP staining) were not investigated, leaving NAC's precise pathways unresolved. Future studies should aim to integrate NAC with conventional endodontic therapy, combining treatment and supplementation for better bone recovery and systemic homeostasis post‐endodontic treatment, alongside immunohistochemical analysis to uncover the mechanisms of action.

## 5 Conclusion

Our results together suggest that systemic administration of N‐acetylcysteine appears to be a promising adjuvant therapeutic strategy to counteract the main pathological mechanisms of apical periodontitis progression since it was able to reduce blood biochemical changes, preserve bone structure, reduce inflammatory infiltrate, and maintain collagen fibres.

## Author Contributions

Deborah Ribeiro Frazão and João Daniel Mendonça de Moura were responsible for conceptualization, methodology, and investigation. Ian Wesley Rocha dos Santos, Deiweson Souza‐Monteiro, Deborah Ribeiro Frazão, Zuleni Alexandre Lisboa da Silva, João Daniel Mendonça de Moura, Jorddy Neves Cruz, and Fabrício Mezzomo Collares contributed to data curation, validation, visualisation, and formal analysis. Deborah Ribeiro Frazão, Ian Wesley Rocha dos Santos, Deiweson Souza‐Monteiro, Zuleni Alexandre Lisboa da Silva, João Daniel Mendonça de Moura, and Jorddy Neves Cruz participated in writing – original draft. Renata Duarte de Souza‐Rodrigues, Luciano Tavares Ângelo Cintra, and Rafael Rodrigues Lima were responsible for writing – review and editing. Renata Duarte de Souza‐Rodrigues and Rafael Rodrigues Lima supervised the work and were also involved in funding acquisition. All authors reviewed and approved the final version of the manuscript.

## Funding

This work was supported by the Conselho Nacional de Desenvolvimento Científico e Tecnológico (CNPq, Brazil) of the Brazilian Ministry of Science, Technology and Innovation through Researcher Productivity Grant (No. 307747/2025‐5 to R.R.L.). Additional support was provided by the Coordenação de Aperfeiçoamento de Pessoal de Nível Superior (CAPES, Brazil), Ministry of Education, under Finance Code 001. R.R.L. also received funding from CNPq through research grants No. 408329/2022‐0, 404431/2024‐0, 400706/2024‐5 and 409576/2025‐5, as well as from the National Institute of Science and Technology in 3D Printing and Advanced Materials Applied to Human and Veterinary Health from CNPq (INCT 3D‐Saúde, No. 406436/2022‐3).

## Ethics Statement

The study was approved by the Ethics Committee on Experimental Animals of the Federal University of Pará (protocol #3160221220).

## Conflicts of Interest

The authors declare no conflicts of interest.

## Supporting information


**Data S1:** iej70108‐sup‐0001‐priase2021‐checklist.docx.

**Data S2:** iej70108‐sup‐0002‐arrive‐checklist‐panac‐final.docx.

## Data Availability

The datasets generated and analysed during this study are available from the corresponding author upon reasonable request.
